# Increasing routine HIV testing in low and medium secure forensic settings

**DOI:** 10.1192/bjo.2021.596

**Published:** 2021-06-18

**Authors:** Rachel Swain, Maja Dujic, Timothy Leung

**Affiliations:** West London NHS Trust

## Abstract

**Aims:**

There remain a number of barriers to patients taking HIV tests, and prevalence of HIV in patients with severe mental illness can be higher than those without. Patients in forensic settings may be at even greater risk. National standards state that in areas of high and extremely high prevalence of HIV, testing should be offered routinely on admission to hospital. A review of compliance with these standards took place across low and medium secure male forensic wards in West London, followed by implementation of targeted interventions to increase testing rates. A reaudit was later completed to assess if changes had resulted in lasting effects

**Method:**

A retrospective review of computer records took place to identify all inpatients residing on the low and medium secure wards on the day of data collection. Their pathology records were checked to ascertain if HIV test results were available. If no test was documented here, then patient psychiatric records were searched for documentation of the test being offered.

After the initial audit, education of patients and staff regarding the benefits of HIV testing took place, HIV testing was incorporated into primary healthcare routine admission screening and separate consent forms were eradicated.

The reaudit took place with data collection occurring in an identical manner.

**Result:**

183 patients were initially identified across 5 low and 7 medium secure male wards, and 184 on reaudit. The initial audit found that only 30.6% (56/183) of patients had either been offered an HIV test or had a result recorded on the pathology system, but this rose to 82.6% (154/184) on reaudit. After the interventions, 43.4% of all patients had HIV test results available, compared to 23.5% initially. Even where no test result existed, the number of tests offered rose from 7.1% to 39.1% of all patients.

**Conclusion:**

This study shows that simple measures to normalise HIV testing and make it part of routine admission screening had dramatic implications for the number of patients being offered an HIV test.

There is still room for improvement, however, with 17.4% of patients having neither test results available, nor documentation that a test was offered. This could be a result of poor general engagement with health care services, and would benefit from thorough documentation and assertive outreach.

